# The Role of the Arcuate Nucleus in Regulating Hunger and Satiety in Prader-Willi Syndrome

**DOI:** 10.3390/cimb47030192

**Published:** 2025-03-14

**Authors:** Charlotte Höybye, Maria Petersson

**Affiliations:** 1 Department of Endocrinology, Karolinska University Hospital, 171 76 Stockholm, Sweden; maria.petersson@ki.se; 2 Department of Molecular Medicine and Surgery, Karolinska Institutet, 171 77 Stockholm, Sweden

**Keywords:** PWS, arcuate nucleus, hyperphagia, oxytocin, orexin, kisspeptin, tachykinins, BDNF, nesfatin-1

## Abstract

Prader-Willi syndrome (PWS) is a rare genetic disorder. The main characteristics are muscular hypotonia, failure to thrive and feeding problems in infancy, which switch to hyperphagia in early childhood and continue into adulthood. Due to hyperphagia, the risk of developing morbid obesity is high without treatment. PWS is considered a hypothalamic disease, and within the hypothalamus the arcuate nucleus (AC) is of central importance for controlling metabolism, hunger, and satiety. The AC has been studied in several animal models as well as in humans, including PWS. The function of AC is regulated by several neuropeptides and proteins produced within the central nervous system such as oxytocin, orexin, tachykinins as well as the hypothalamic hormones, regulating the adeno-hypophyseal hormones, also acting as neurotransmitters. Additionally, there are many peripheral hormones among which insulin, leptin, adiponectin, ghrelin, and glucagon-like peptide (GLP-1) are the most important. High levels of adiponectin and ghrelin have consistently been reported in PWS, but dysregulation and deviating levels of many other factors and hormones have also been demonstrated in both individuals with PWS and in animal models. In this review, we focus on the role of AC and peptides and proteins produced within the central nervous system in the regulation of hunger and satiety in PWS.

## 1. Introduction

Prader-Willi syndrome (PWS) is a rare, multi-symptomatic, contiguous genetic disorder [1]. PWS is caused by absence of paternally expressed imprinted genes at 15q11.2-q13 through paternal deletion of this region (65–75% of individuals), maternal uniparental disomy 15 (20–30%), or an imprinting defect (1–3%) [1]. The estimated prevalence is 1/10,000-1/21,000 newborns [2].

PWS is considered a hypothalamic disease and many of the symptoms of the syndrome are related to hypothalamic dysfunction. The syndrome is classically characterized by muscular hypotonia, poor growth, and short stature, with feeding problems in infancy replaced by hyperphagia at approximately 4 years of age leading to a high risk of obesity, unless food intake is supervised and controlled [3]. Body composition is abnormal with more body fat than muscle mass, and metabolism is decreased. Behavioral problems including temper outbursts, anxiety, obsessive-compulsive and autism-like features, as well as cognitive and learning disabilities, are common. Endocrine abnormalities are commonly observed, with insufficient growth hormone (GH) secretion, and hypogonadism being most frequently present. The knowledge of PWS and clinical management has improved considerably over recent decades. Together with the approval of GH treatment of children with PWS from the year 2000, it has led to a marked change in the phenotype, with normal adult height and improved psychomotor functioning and body composition [3].

Several of the genes in the affected region of PWS have been studied, and dysfunction of each of them can be associated with one or several of the symptoms of PWS [4]. However, it has not been possible to relate the symptoms of PWS to any single causative gene, and the symptoms are more likely to result from the entire genetic deletion [4]. RNA sequencing of the hypothalamus from individuals with PWS showed that upregulated genes overlapped with the mouse agouti-related peptide (AgRP) neurons, mainly located within the AC and activated by hunger, while downregulated genes overlapped with the expression profile of proopiomelanocortin (POMC) neurons, which in the AC are activated by feeding and satiety [5].

Magnetic resonance (MR) imaging studies have shown that all hypothalamic nuclei are smaller in adults with PWS compared to both age and gender-matched controls as well as participants with obesity [6]. Also, in children with PWS, MR imaging has shown atrophy in the thalamus, pallidum, hippocampus, amygdala, and hypothalamus compared to obese, age- and sex-matched controls [7]. Further, premeal functional MR imaging demonstrated higher activity in reward/limbic regions (nucleus accumbens, amygdala) and lower activity in the hypothalamus and hippocampus in response to food vs. non-food in individuals with PWS compared to participants with obesity [8]. Post-meal, individuals with PWS exhibited a greater stimulation of food activation centers in the limbic and paralimbic regions (hypothalamus, amygdala, hippocampus) and a lower activation in cortical inhibitory areas (orbitofrontal cortex, medial prefrontal cortex) [9].

Thus, knowledge of the function of the hypothalamus is important for understanding metabolism and the regulation of hunger and satiety in PWS. This review summarizes the central regulation with a focus on the arcuate nucleus (AC) and neuropeptides and proteins produced within the central nervous system (CNS) and especially within the hypothalamus in PWS.

## 2. The Role of the Hypothalamus in Appetite and Metabolism

The hypothalamus is central in controlling metabolism, appetite, thirst, temperature, diurnal rhythm, and hormonal secretion from the pituitary. It is located in the basal part of the brain between the thalamus and the pituitary and integrates and transmits signals from other parts of the brain, the autonomic nervous system, and from hormones and peptides from the periphery. Although the regulation of hunger and satiety is controlled from more than one hypothalamic nucleus, the AC is often considered the most important. The AC has a reciprocal connection with the other hypothalamic nuclei involved in the regulation of hunger and satiety, such as the paraventricular nucleus (PVN), where for example, oxytocin and corticotrophin-releasing hormone (CRH) are produced; the hypothalamic lateral nucleus, which contains many orexin neurons and is considered especially important for hunger; and the ventromedial nucleus, which is considered especially important for satiety.

The hypothalamus, and in particular the AC, have close connections to the mesolimbic system, where dopamine and serotonin are two of the main neurotransmitters. Studies have shown that individuals with PWS also may have deviations in these pathways and neurotransmitters [4,10].

### 2.1. The Role of the Arcuate Nucleus in Appetite and Metabolism

The AC is located in the mediobasal part of the hypothalamus close to the third ventricle and the median eminence. This nucleus contains two specific neuron populations: the neuropeptide Y (NPY) and AgRP neurons and the POMC cocaine and amphetamine-regulated transcript (CART) neurons. The NPY/AgRP neurons stimulate appetite and the POMC/CART-neurons are stimulated by feeding/satiety and inhibit appetite.

The two neuron populations also interact with each other; for example, there is γ-aminobutyric acid (GABA)-ergic inhibition of the POMC/CART neurons from the NPY/AgRP neurons of the POMC. The NPY/AgRP and POMC/CART neurons both express receptors for many important hormones and neurotransmitters that regulate their activity. For example, leptin, produced mainly in the white adipose tissue, decreases the activity in the NPY/AgRP neurons and increases the activity in the POMC/CART neurons. Similar effects are induced by insulin. Thus, both leptin and insulin stimulate satiety. However, leptin and insulin resistance are common in obesity, where the effects of leptin and insulin within the AC, and especially in the POMC/CART neurons, become less pronounced and sometimes these effects may even be absent [11,12]. However, in this context, it should be noted that individuals with PWS have less insulin resistance and lower insulin levels compared to obese individuals without PWS [13]. One mechanism behind this might be the higher adiponectin levels that have been reported in PWS [14].

Other hormones important for the activity of these neurons in the AC are ghrelin, GH, peptide YY (PYY), orexin, GLP-1, oxytocin, kisspeptin, brain-derived neutrophic factor (BDNF), asprosin, as well as the hypothalamic-pituitary-adrenal (HPA)-axis.

**Figure 1:**
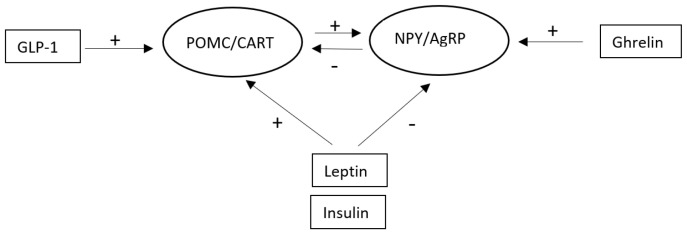
Some of the most important gastrointestinal and adipose tissue hormones regulating neurons in the arcuate nucleus (+ indicates an increased activity in neurons, − indicates a decreased activity in the neurons). Abbreviations: AgRP: agouti-related peptide, CART: cocaine and amphetamine-regulated transcript, GLP-1: glucagon-like peptide 1, NPY: neuropeptide Y, POMC: proopiomelanocortin.

Some of the most important gastrointestinal and adipose tissue hormones regulating neurons in the AC are shown in Figure 1.

### 2.2. POMC/CART Neurons

POMC undergoes cleavage into α-MSH that binds to melanocortin (MC) receptors, preferably MC4 receptors, thereby activating satiety. The POMC/CART neurons are regulated primarily by leptin, insulin, and glucose levels but also by GLP-1, which all suppress food intake. Stimulation of POMC/CART neurons in mice reduces food intake and body weight [15]. POMC knockout mice develop hyperphagia, insulin resistance, and obesity. In addition, CART knockout mice exhibit the same characteristics [12].

### 2.3. NPY/AgRP Neurons

The NPY/AgRP neurons are stimulated by ghrelin and inhibited by leptin, insulin, and glucose acting through the stimulation of NPY receptors and inhibition of MCRs (for reviews see [11,12]). In addition, CRH may both directly, and indirectly through other peptides and neurotransmitters, influence the NPY/AgRP neurons and has been suggested to have a role in stress-related eating behavior [16,17]. Both NPY and AgRP knockout mice have unchanged weight and food intake. In contrast, transgenic mice overexpressing AgRP develop obesity. So do NPY receptor knockouts, although food intake seems to be unaltered [18,19,20,21].

### 2.4. Other Neurons in the Arcuate Nucleus

The NPY/AGRP and the POMC/CART neurons are not the only neurons in the AC. There are, for example, neurons producing GABA and dopamine, where dopamine is released both as a neurotransmitter and as a neurocrine regulator of prolactin. Additionally, several neuropeptides and proteins such as GHRH, kisspeptin, tachykinins, somatostatin, and GnRH are also synthesized within the arcuate nucleus [11].

## 3. The Arcuate Nucleus in PWS

Dysfunction of the AC is involved in several states and disorders related to overeating, weight and growth diversities, metabolism, and obesity. Therefore, the AC is highly interesting in PWS; and as mentioned above, individuals with PWS generally have a smaller AC [6].

The 15q11-13 region contains both protein-coding DNA genes and differentially imprinted genes. Mutations or deletions of genes in this region (for example *MAGEL2*, *NECDIN*, and *SNORD116*) are in mouse models associated with hypotonia, developmental delay, hypogonadism, hyperphagia, and impaired social skills [22].

The *MAGEL2* gene is expressed in the POMC neurons in the AC of the hypothalamus; knockout of the *MAGEL2* gene disrupts POMC neuronal circuits and functions in rodents and they become obese. A change in the sensitivity and stability of leptin receptors has been suggested to be one of the mechanisms behind this effect [23,24]. In a study of leptin sensitivity in *MAGEL2* gene-knockouts, there was a gradual decrease in leptin sensitivity, such that POMC neurons responded normally to leptin in newborn rats up to 4 weeks of age, whereas the response was reduced at the age of 6 weeks [25].

Contrary to what is expected, the Necdin knockout mice do not usually exhibit obesity. Studies have instead shown that these mice often have lower body weight and a reduced amount of adipose tissue [26].

Neither do *SNORD116* knockout mice typically exhibit obesity. These mice are often born smaller and remain smaller than their wild-type counterparts throughout their lives. However, when *SNORD116* is deleted in adult mice, they do display hyperphagia (increased food intake). Interestingly, only some of these adult knockout mice become obese, and the reason behind this is to our knowledge not known [27].

In PWS, the expression of the transcription factor nescient helix-loop-helix 2 (*NHLH2*) is reduced and *NHLH2*-knockout mice are obese. Indeed, these mice have reduced mRNA levels of both POMC and CART and increased levels of NPY and AGRP [28]. NHLH2 is also associated with kisspeptin and the *NHLH2* knockouts exhibit hypogonadism [29].

In a transgenic mouse model of PWS, which displayed failure to thrive during the neonatal period, the expression of AgRP mRNA was decreased while the mRNA expression of POMC was upregulated in PWS-mice neonates. Since AgRP stimulates appetite and the POMC-derived peptide, α-MSH, stimulates satiety, these changes together will decrease feeding and may thus contribute to the failure to thrive in PWS neonatal mice [30]. These changes are consistent with the findings in Magel 2 knockouts described above and also with the different periods in PWS, in which feeding difficulties in infants change to overeating after a couple of years.

In a study of hypothalamic tissue from individuals with PWS, it was demonstrated that upregulated genes overlapped with rodent RNA-sequencing data from the AgRP/NPYneurons, whereas downregulated genes overlapped with rodent RNA corresponding to the POMC/CART neurons [5]. However, in another study on post-mortem hypothalamic tissues, Goldstone et al. reported unchanged expression of AgRP as well as NPY in PWS [31].

In children with PWS, serum levels of α-MSH were significantly lower compared to obese and lean controls. No difference in AgRP levels was seen [32]. In contrast to what would be expected, the number of NPY neurons are reduced in PWS, maybe because of increased levels of leptin and insulin [4]. NPY in plasma is mainly derived from the sympathetic nervous system, the adrenal gland, and other tissues in the periphery, and does not reflect NPY levels within the brain. Usually, there is no change in plasma levels of NPY in individuals with PWS, although data on NPY are conflicting [13]. Ghrelin levels are increased in individuals with PWS compared to both lean controls and obese subjects [4,33], and as discussed above, ghrelin increases the release of NPY and AgRP from the AC. Snord116 knockout mice have increased ghrelin levels as well [34].

Leptin levels in PWS relate to the amount of adipose tissue in the same way as in obese non-PWS individuals [35], whereas the prevalence of impaired glucose intolerance, hyperinsulinemia, and type 2 diabetes seems to be lower compared to that in obese non-PWS controls [20]. Adiponectin, a peptide that also is produced within the adipose tissue, is mainly associated with positive metabolic effects such as decreased insulin resistance.

Adiponectin receptors are expressed both in the POMC/CART and the NPY/AgRP neurons and increased levels are mainly associated with satiety. Indeed, PWS individuals have been demonstrated to have higher adiponectin levels compared to obese non-PWS controls.

It is important to recognize that there are different genetic mechanisms that can be responsible for PWS, and it is not surprising that there are conflicting findings, since the genotype in the study cohorts were probably not the same type. Even then, other modulatory genes exist so that not everyone would exhibit the same symptoms in every aspect.

## 4. How Peptides and Proteins Produced Within the Brain Affect the Arcuate Nucleus—And Their Potential Roles in PWS

### 4.1. Oxytocin

Oxytocin is produced within two hypothalamic nuclei, the PVN and the supraoptical nucleus. Oxytocin is released as a hormone from the neurohypophysis and is mainly associated with its hormonal effects during parturition and breastfeeding. However, it is also released within the brain and involved in, for example, behavior and appetite regulation. Oxytocin has anxiolytic-like effects and it may both increase and decrease food intake depending on physiological context, other hormones, and if in a fed or fasting state [36,37]. There are oxytocin receptors on both the POMC/CART neurons and the NPY/AgRP neurons. Analyses of tissue from the PVN of adults with PWS showed a reduction of both the volume, the cell number, and the immunoreactivity of oxytocin neurons, and a reduction of oxytocin has been suggested to be one of the mechanisms behind the hunger in PWS [38,39]. In contrast, analyses of oxytocin in cerebrospinal fluid showed higher concentrations in adolescents and adults with PWS compared to the controls [40]. In line with this, plasma levels of oxytocin in 23 children with PWS were compared with those of 18 healthy unrelated siblings matched for age and with a similar gender ratio and BMI. The children with PWS were found to have levels of plasma oxytocin more than twice as high compared to those of unrelated siblings [41]. However, analyses of serum oxytocin in adults with PWS showed similar concentrations as in controls but, in relation to their obesity, the concentrations were low [35]. Interestingly, oxytocin is released in response to vagal stimulation [42] and clinical trials of vagal stimulation in PWS individuals have been conducted with positive results [43]. A pronounced dysregulation of the signaling pathways for oxytocin has been shown in the Magel2-knockout mouse model of PWS. Early postnatal treatment with oxytocin restored behavioral changes that have been observed in these mice [44]. In addition, the initial feeding difficulties in the newborn mice were prevented [45]. Consistent with these observations, studies with oxytocin treatment in individuals with PWS have shown improvements in anxiety, compulsiveness, and hyperphagia. However, the degree of oxytocin’s effects depends on situation and context and seems to be more pronounced if administered early in life [46].

### 4.2. Brain-Derived Neurotrophic Factor

The neurotrophin brain-derived neurotrophic factor (BDNF), synthesized in several brain areas, is besides regulating growth involved in the regulation of energy homeostasis, and may directly affect the NPY/AgRP neurons and inhibit their activity, thus acting as a satiety signal. In addition, BDNF injected intracerebroventricularly as well as directly into the PVN decreased food intake and weight gain in rats [47]. In line with this, a study of a *MAGEL2*-null mouse model of PWS assessed the translational potential of hypothalamic adeno-associated virus (AAV)-BDNF gene therapy and the authors found that BDNF gene therapy improved glucose metabolism, insulin sensitivity, circulating adipokine levels, and body composition [48]. Furthermore, in hypothalamic tissue from deceased individuals with PWS, the number of neurons in the hypothalamus expressing BDNF and *NTRK2*, the gene coding for one of its receptors, were found to be lower [5]. Reduced basal circulating BDNF levels and a diminished postprandial peak in comparison to weight-matched controls have also been demonstrated in individuals with PWS [49].

### 4.3. Orexin

Orexin, which is produced within the hypothalamus, is involved in appetite regulation, arousal, sleep and wakefulness, metabolism and energy expenditure, pleasure and reward-seeking behavior, as well as stress response. There are 2 forms, orexin A and orexin B, which both are important for appetite regulation, but their affinity for the two orexin receptors is different. Changes in peripheral metabolic signals (i.e., glucose, ghrelin, leptin, and blood pH) impact the activity of orexin A neurons in the lateral hypothalamus, which increases eating behaviors through binding to orexin receptors in the hypothalamus, hippocampus, locus coeruleus, and limbic centers. Both the POMC/CART neurons and the NPY/AgRP neurons are provided with orexin receptors. The *MAGEL2*-null mouse has reduced orexin levels as well as orexin neurons [50]. In a study of 23 children with PWS, circulating orexin A levels were found to be higher compared to age-matched controls, suggesting that dysregulation of orexin A signaling might contribute to behavioral problems and hyperphagia in PWS [51]. However, another study of 14 individual with PWS aged 8 to 37 years showed moderately decreased orexin levels in cerebrospinal fluid [52]. There was no correlation between orexin and BMI. The discrepancy between the findings in the two studies might be due to measurements in different fluids and different ages of the patients suggesting involvement of other hormones, such as leptin and ghrelin [53].

### 4.4. Kisspeptin

Kisspeptin is produced within the hypothalamus, including the AC. It is mainly associated with the onset of puberty and the release of GnRH and is thought to play an important role in reproductive function [54]. However, it has also been demonstrated to modulate the release of GH as well as the activity within the AC, where it stimulates POMC/CART neurons, and it may also inhibit the NPY/AGRP neurons. These effects in the AC both decrease appetite. NHLH2, which is reduced in PWS, takes part in the regulation of the effects of kisspeptin levels. Due to the frequently present hypogonadism in PWS, kisspeptin levels would be expected to be low. Unexpectedly, kisspeptin levels have been demonstrated to be higher in individuals with PWS but are normalized in response to GH treatment [52], which might indicate that the raised kisspeptin levels mainly are due to the GH deficiency.

### 4.5. Tachykinins

Tachykinins are a large group of neuropeptides including neurokinin A and B, and substance P. Tachykinins are produced both in the periphery and in the brain and are, for example, involved in pain, vascular tone, and behavior. Neurokinin synthesis is also colocalized with the synthesis of kisspeptin in the so-called KNDy (kisspeptin, neurokinin, dynorphin) neurons within the AC. These neurons are of central importance for the regulation of GnRH. However, neurokinins and substance P also modulate the POMC/CART neurons and tachykinin receptors have been demonstrated in these neurons.

In PWS individuals, plasma levels of substance P as well as beta-endorphin (which is cleaved from POMC) have been found to be increased. These changes have been suggested to contribute to hunger in PWS [55].

### 4.6. Nesfatin-1

Nesfatin-1 is a peptide originating from the precursor molecule nucleobindin-2 and produced in various brain areas including the hypothalamus and the AC. It is also synthesized, for example, within the adipose tissue, the gastrointestinal canal, and the pancreas and it crosses the blood–brain barrier. Serum levels increase in response to feeding and have been demonstrated to inhibit food intake and in addition directly inhibit NPY/AgRP neurons within the AC. Nesfatin-1 has also been demonstrated to increase both oxytocin and CRH (for a review, see [56]). In a recent study of non-obese children with PWS treated with GH, nesfatin-1 levels were higher when compared to age-matched healthy controls [57]. Thus, a change in nesfatin-1 levels might also contribute to the metabolic effects of GH treatment in PWS.

### 4.7. CRH

CRH is mostly produced within the hypothalamic nucleus PVN and is the main regulator of the HPA-axis. CRH is also produced in the amygdala, and similarly to oxytocin CRH, both from the PVN and the amygdala, is released within the brain, acting as a neurotransmitter. CRH is mainly associated with satiety but during certain circumstances it may also increase the activity of NPY/AgRP neurons, thereby increasing hunger. However, the activity within the HPA-axis seems not to be increased in PWS. Instead, a delayed stress response in children with PWS has been demonstrated [58]. However, this does not exclude deviations within the brain CRH release and pathways. In a study with MRI, an increased activation of the amygdala in response to food odors was seen in adult individuals with PWS [59].

### 4.8. TRH

TRH is also mainly produced within the PVN and it is the main regulatory hormone in the thyroid axis. Similarly to CRH, it also acts as a neurotransmitter. During starvation and/or long-term illness, pathways from the NPY/AgRP neurons in the AC decrease the release of TRH. There is also a TRH pathway from the PVN to the AC and TRH receptors in the NPY/AgRP neurons, which have been suggested to be excitatory, and thus it would be expected that they should increase hunger [60]. Hypothyreosis is common in PWS and the most common cause is central hypothyroidism, probably caused by a deficiency or dysregulation of TRH [3].

### 4.9. GHRH

GHRH is produced within the AC and is necessary for the stimulation of growth hormone (GH) release from the pituitary. This release of GH may then modulate the POMC/CART and NPY/AGRP neurons, which both express GH receptors. GH is also released by ghrelin. Individuals with PWS have deficient GH secretion, and leptin levels decrease and body composition improves in response to GH treatment. Worth mentioning is that adiponectin levels do not change in response to GH treatment [35]. GH treatment of individuals with PWS might, through negative feedback, induce the already low GHRH release to become even lower. However, although GHRH is produced within the AC, there are, to our knowledge, no GHRH receptors on the POMC/CART or NPY/AgRP neurons.

## 5. Discussion

The regulation of hunger and satiety is very complex, and several genes and transcription factors, as well as central and peripheral secreted hormones, neuropeptides, and proteins are involved. In PWS, the dysfunction of the hypothalamus, hyperphagia, abnormal body composition, and obesity make it even more complicated. Within the hypothalamus, the AC is of central importance for metabolism, hunger, and satiety. The focus of our review was the effects of brain-derived peptides and proteins on the AC, and in summary, we found that several of these substances have the potential to disturb the function of the NPY/AgRP and POMC/CART circuits in PWS, resulting in unbalanced systems.

In the present review, we have discussed oxytocin, BDNF, orexin, kisspeptin, tachykinins, nesfatin-1, as well as the hypothalamic hormones and neurotransmitters CRH and TRH, and all of them seem to be dysregulated in individuals with PWS (Table 1). In addition, the release of GHRH is decreased, thus mediating a lower release of GH, which in turn may affect the regulation of the POMC/CART and NPY/AgRP neurons. Indeed, there are improvements in leptin, insulin, and kisspeptin levels in response to GH treatment. The AC is, of course, not the only nucleus within the hypothalamus that regulates appetite and satiety. The PVN, where, for example, oxytocin, CRH, and TRH are produced, also participates in this integrated regulation, as does the ventromedial hypothalamic nucleus (VMH), sometimes considered as the hypothalamic satiety center and the lateral hypothalamic nucleus (LH), sometimes considered as the hypothalamic hunger center. All these nuclei communicate bidirectionally with each other.

There are other factors besides the peptides and proteins discussed in the present paper, including the classical neurotransmitters and also lipids. Endocannabinoids are produced in many peripheral organs and tissues as well as the brain and the hypothalamus. There are cannabinoid receptors in the AC and in general they are considered as appetite stimulators.

Indeed, the activity of cannabinoids has been demonstrated to be increased in PWS. The *MAGEL2* knockout mice discussed earlier in this manuscript have been found to have increased activity within their endocannabinoid systems as well. In clinical studies where a cannabinoid receptor 1-antagonist was given to PWS individuals, weight was reduced [61]. However, clinical trials with cannabinoid receptor antagonists have been terminated because of unwanted side effects.

Besides these centrally released factors, there are several hormones produced in the adipose tissue, the pancreas, and the gastrointestinal tract, which also participate in the regulation of the AC in addition to the previously mentioned hormones: leptin, insulin, GLP-1, and ghrelin. Asprosin was discovered in 2016 and is a protein produced in the adipose tissue. Asprosin participates in the regulation of blood glucose levels through stimulating liver glucose production and appetite [62]. In the AC, asprosin may inhibit the POMC/CART neurons, which is thought to be one of the main mechanisms behind its appetite stimulating effect. In a recent study by Faienza et al. [63], obese individuals with PWS had higher asprosin levels when compared to PWS with normal weight. Asprosin levels were also higher in adults compared to adolescents. Interestingly, asprosin levels were significantly higher in patients with deletion versus patients with uniparental disomy. However, no correlation between serum levels of asprosin and appetite in individuals with PWS could be demonstrated. Other peripheral hormones with receptors in the brain may also contribute to changed appetite and eating behavior. Both cholecystokinin (CCK) and PYY levels are lower in individuals with PWS, and these hormones are associated with satiety and increase in response to feeding. In contrast, fasting and postprandial GLP-1 levels have been reported to be similar between individuals with PWS and obese and lean controls [64].

As already mentioned, ghrelin levels are increased in PWS individuals. Animal studies indicate that ghrelin is produced not only in the gastrointestinal tract and islets of Langerhans but also within the hypothalamus [65,66]. Prior to secretion, ghrelin is acetylated (AG), and in this form, it activates the GH secretagogue receptor 1a (GHSR1a), and the release of GH. Adrenocorticotropic hormone (ACTH), cortisol, prolactin, and glucose are also released in response to AG and AG affects feeding, growth, fat stores, and glycemic regulation. Unacetylated ghrelin is converted to AG by the enzyme ghrelin O-acyltransferase (GOAT). GLWL-01 is a selective, reversible inhibitor of GOAT, and in a double-blind, placebo-controlled, phase 2 study, GLWL-01 was found to lower the AG levels and the ratio of AG/UAG [67]. However, no effects on hyperphagia or on anthropometric and clinical parameters related to weight excess in patients with PWS were observed, maybe due to a short duration of treatment and a small study cohort.

UAG inhibits AG-induced hunger, reduces fat deposition, and improves insulin resistance. In studies with livoletide, an UAG analog, no significant change was found in hunger and food-related behaviors as measured with the Hyperphagia Questionnaire for Clinical Trials (HQ-CT) scores, or in fat mass, body weight, or waist circumference compared with placebo [68].

Likewise, studies on setmelanotide, a melanocortin (MC)-4 receptor agonist administrated subcutaneously once daily, showed no changes in weight, DEXA measurements or laboratory findings, while the mean hyperphagia questionnaire scores demonstrated a small, not statistically significant reduction from baseline. Further studies with setmelanotide have not been performed [69].

Ghrelin has, besides its direct effects in the AC, also been demonstrated to increase appetite by acting through dopamine, serotonin, opioid, and cannabinoid systems. We have already discussed the cannabinoid system above but the neurotransmitters have mainly been left aside in the present paper. Worth mentioning here, however, is that serotonin, and especially the 5HT2c receptors, are central in hypothalamic appetite regulation including the AC. Alternate splicing of the serotonin (5-hydroxytryptamine; 5-HT) 2C receptor (5-HT_2C_R) pre-RNA is negatively regulated by *Snord115* [70,71], and loss of *Snord115* expression is associated with decreased levels of POMC in the AC [71].

Recently, clinical trials with diazoxide, a drug commonly used to treat hypoglycemia and hyperinsulinemia, have been performed. Diazoxide is a potent activator of the adenosine triphosphate-sensitive potassium (KATP) channel and crosses the blood–brain barrier [72]. Activation of the KATP channel in NPY/AgRP neurons in the hypothalamus reduces the secretion of NPY and AgRP [73]. In addition, the KATP channels in the dorsal motor nucleus of the vagal nerve, pancreatic β-cells, and adipocytes are activated, reducing hyperinsulinemia and body fat, directly or indirectly leading to improved insulin and leptin resistance and satiety [73]. These effects have been confirmed in animal models of hyperphagic obesity including Magel-2 null mouse [73].

Diazoxide choline slow-release (DCCR) treatment has in a phase 2 study in individuals with PWS been shown to reduce hyperphagia, aggressive behavior, and body fat, and increase lean body mass [74]. In a following 13 week phase 3 study comparing DCCR to placebo, DCCR improved body composition and clinician-reported outcomes, but did not in the entire cohort significantly improve hyperphagia in the primary analysis. However, DCCR reduced hyperphagia in those with more severe hyperphagia, and in the subset of data collected before the onset of the COVID-19 pandemic [75]. In the open-label extension of the phase 3 study, 125 participants with PWS received DCCR for up to 52 weeks [76]. This study showed that DCCR improved hyperphagia, with greater improvements in those with more severe baseline hyperphagia. Improvements were also seen in aggression, anxiety, and compulsivity, and there were reductions in leptin, insulin, and insulin resistance, as well as a significant increase in adiponectin.

Although patients with PWS are similar in certain aspects, individuals’ manifestations may differ depending on the genotype and other modifier genes. Most well-known is the increased risk of psychosis and behavioral challenges in the UPD subgroup. However, it has also been shown that the frequency and severity of hyperphagia and obesity are more prominent in the deletion group compared to the UPD group [77]. Differentially expressed genes associated with PWS are *MKRN3, MAGEL2, NECDIN*, and small nucleolar RNA genes, and abnormalities such as UPD of maternal alleles result in a lack of paternally expressed genes. *SNORD116*, a small nucleolar RNA gene, is known to be the critical gene for most PWS phenotypes, and deletion of this gene has been demonstrated to cause an imbalance in the neuromodulatory systems of the hypothalamus, and results in hyperphagic behavior and sleep disturbances. Studies generating specific information on the function of the AC in deletion and UPD groups is not yet available but would be expected in the future.

## 6. Conclusions

In conclusion, the AC plays an important role in the very complex and multifactorial regulation of hunger and satiety. Our knowledge is primarily based on animal studies and measurements of circulating levels of various neuropeptides and hormones, which are usually not parallel with CNS levels. In individuals with PWS and in animal models of PWS, hormones such as oxytocin, ghrelin, and adiponectin seem to be secreted and to circulate in different concentrations compared to those in non-PWS humans or animals. Other factors such as deficiency of adenohypophyseal hormones, as well as an abnormal body composition, less insulin resistance, and comorbidities, also affect the outcome. In line with this, some clinical trials in PWS with specific pharmacological compounds important for the regulation of hunger and satiety have been without significant effects. The regulation appears to be multifactorial, involving a complicated interplay between different factors, making it difficult to identify a specific neuropeptide or hormone as the most crucial in PWS. However, promising results have been obtained with treatments using diazoxide and oxytocin and further investigations of the regulation of hunger and satiety in PWS are ongoing.

## Figures and Tables

**Table 1:** Changes in peptides and proteins produced within the hypothalamus in animal models or individuals with PWS.

Peptide/Protein	Difference	Type of Study	References
Oxytocin	Usually low	Autopsy material, human blood, and CSF as well as animals	[35,38,39,40,41,44]
Orexin	High	Human blood and anjmals	[50,51]
Kisspeptin	High	Human blood	[52]
BDNF	Low	Autopsy material, animals, and human blood	[5,48,49]
Tachykinins	Substance P high	Human blood	[55]
Nesfatin-1	High	Human blood	[57]
CRH	Changed activity	Human blood, MR	[58,59]
TRH	Low	Human blood	[3]
GHRH	Low	Human blood	[3]

Abbreviations: BDNF, brain-derived neurotrophic factor; CRH, corticotrophin releasing factor; GHRH, growth hormone releasing hormone; TRH, thyrotropin releasing hormone.
